# Pooled-Peptide Epitope Mapping Strategies Are Efficient and Highly Sensitive: An Evaluation of Methods for Identifying Human T Cell Epitope Specificities in Large-Scale HIV Vaccine Efficacy Trials

**DOI:** 10.1371/journal.pone.0147812

**Published:** 2016-02-10

**Authors:** Andrew Fiore-Gartland, Bryce A. Manso, David P. Friedrich, Erin E. Gabriel, Greg Finak, Zoe Moodie, Tomer Hertz, Stephen C. De Rosa, Nicole Frahm, Peter B. Gilbert, M. Juliana McElrath

**Affiliations:** 1 Vaccine and Infectious Disease Division, Fred Hutchinson Cancer Research Center, Seattle, Washington, 98109, United States of America; 2 Biostatistics Research Branch, National Institute of Allergy and Infectious Disease, Rockville, Maryland, 20852, United States of America; 3 Shraga Segal Department of Microbiology, Immunology and Genetics, Ben Gurion Institute of the Negev, Beer-Sheva, 84105, Israel; University of Massachusetts Medical Center, UNITED STATES

## Abstract

The interferon gamma, enzyme-linked immunospot (IFN-γ ELISpot) assay is widely used to identify viral antigen-specific T cells is frequently employed to quantify T cell responses in HIV vaccine studies. It can be used to define T cell epitope specificities using panels of peptide antigens, but with sample and cost constraints there is a critical need to improve the efficiency of epitope mapping for large and variable pathogens. We evaluated two epitope mapping strategies, based on group testing, for their ability to identify vaccine-induced T-cells from participants in the Step HIV-1 vaccine efficacy trial, and compared the findings to an approach of assaying each peptide individually. The group testing strategies reduced the number of assays required by >7-fold without significantly altering the accuracy of T-cell breadth estimates. Assays of small pools containing 7–30 peptides were highly sensitive and effective at detecting single positive peptides as well as summating responses to multiple peptides. Also, assays with a single 15-mer peptide, containing an identified epitope, did not always elicit a response providing validation that 15-mer peptides are not optimal antigens for detecting CD8+ T cells. Our findings further validate pooling-based epitope mapping strategies, which are critical for characterizing vaccine-induced T-cell responses and more broadly for informing iterative vaccine design. We also show ways to improve their application with computational peptide:MHC binding predictors that can accurately identify the optimal epitope within a 15-mer peptide and within a pool of 15-mer peptides.

## Introduction

Measures of immunogenicity are an integral part of iterative and rational vaccine design [1,2].

The enzyme-linked immunospot (ELISpot) assay can be used to quantify the frequency of antigen-specific T cells via the secretion of interferon gamma (IFN-γ) following brief *ex vivo* stimulation with one or multiple peptide antigens [3,4]. The magnitude readout of the assay is the frequency of cytokine-secreting cells (spot-forming cells per million [SFC/M]), and although IFN-γ itself is not necessarily linked to CD8+/CD4+ T cell cytolytic activity [5], ELISpot responses identify antigen-specific T cells and have previously been associated with endpoints in vaccine clinical trials [6–11]. One of the main advantages of the ELISpot assay over flow-cytometric assays (e.g., intracellular cytokine staining [ICS] [12] or tetramer sorting) is the ability to efficiently screen a wide array of peptide antigens covering the entire set of vaccine immunogens, thus effectively mapping the specificity of T-cell responses [13]. Another benefit is that extensive effort has gone towards standardization of the assay across labs to reduce variability [14–16], and towards statistical method development, which has improved positivity calls [2,17–22].

Much of the work to develop and optimize the assay was motivated by a need to quantify vaccine-induced T-cell responses in HIV vaccine trials. Based on studies establishing associations between HLA class I alleles and slower progression to AIDS [23–27], it is hypothesized that vaccine-primed T cells may slow disease progression if breakthrough infection occurs [28]. To date, most candidate HIV vaccines have elicited HIV-specific T-cell responses [29–33] including the recombinant adenovirus-vectored vaccine tested in the Step Study, in which vaccine recipient responders targeting at least three Gag epitopes had lower viral load compared to non-responders [11].

Mapping the specificities of vaccine-induced responses is necessary for a rational approach to vaccine design, since T-cell responses vary in their effect on HIV disease progression [34–38]. Identifying vaccine-induced T-cell epitopes is made easier by the fact that the sequence of the vaccine immunogen is known. This greatly restricts the number of antigens that need to be tested as potential epitopes in “epitope mapping” assays. However, it is also of great importance to measure the responses of vaccine-primed T cells to circulating viral variants (i.e., response “depth”). This requires testing of many additional potential antigens [39] and will be made more complicated as HIV vaccines are designed to provide coverage of multiple subtypes [40]. Epitope mapping of vaccine-induced responses can be more challenging as they are typically lower in magnitude than those observed in natural infection [16]. However, recently Borthwick et al. showed that prime-boost regimens of adenovirus- and pox virus-vectored vaccines containing conserved elements of HIV elicited broad and high magnitude T-cell responses, demonstrating that it is possible to elicit robust cellular responses with a vaccine and that they can be readily detected using ELISpot [41].

Several methods for epitope mapping have been devised to efficiently utilize participant samples and other lab resources. Since typically only a few peptides elicit a T-cell response in vaccine studies, “group testing” approaches have been developed where pools of peptides are evaluated in one assay; peptides in pools that don’t elicit a response can be quickly eliminated *en masse* [16,42–45]. Pools can be designed in several hierarchical stages of decreasing size until individual peptides are identified. Pools can also be used in a matrix-based strategy such that each column and row in a matrix of peptides is tested together. This specific arrangement, such that each peptide is in exactly one row-pool and one column-pool, allows for more efficient identification of responses at the “intersection” of positive pools. Ultimately, in any strategy a positive response must be confirmed using single peptide stimulation. The sizes and design of peptide pools can be optimized based on the expected number of responses per participant and the expected covariation of response across the cohort [43,44,46]. Though the theoretical efficiencies, sensitivities and specificities have been computed for many group testing algorithms, the calculations require assumptions about the sensitivity and specificity of the assay that may not hold in practice. For example, specificity for any single antigen may decrease for pools with a large number of peptides, or pool sensitivity may increase when they contain multiple positive peptides. For this reason it is important to study the operating characteristics of group testing procedures experimentally.

In this study we evaluated two common group testing based strategies for epitope mapping using previously cryopreserved PBMC samples from participants in the Step Study. We compared these methods to each other and to a “Test-all” method in which every peptide was tested individually. Though the Test-all method would be too costly in practice, by testing individual peptides it identified the greatest number of epitopes and provided a standard by which to evaluate the two group testing strategies. We found that the group testing strategies reduced the number of ELISpot assays that were required by as much as 7.7-fold and did not result in significantly lower accuracy in estimating breadth. Assays using small pools of peptides (7–30 peptides) were highly sensitive and were able to detect responses to single positive peptides, as well as summate responses to multiple peptides. The overlapping peptides were an efficient way to map T-cell epitopes; however, some HLA class I restricted T cells may require shorter optimal-length peptides for robust stimulation. These findings provide experimental validation of group testing-based strategies for identifying vaccine-induced T-cell epitopes. The results also provide further motivation to continue optimization and standardization of epitope mapping methods in vaccine trials, which will be critical for the evaluation and comparison of novel vaccine regimens.

## Materials and Methods

### Study cohort

The Step Study (Merck V520-023/HVTN 502) was a multicenter, double-blind, randomized, placebo-controlled phase IIb proof-of-concept study to evaluate the safety and efficacy of the MRKAd5 HIV Gag/Pol/Nef trivalent vaccine in 3,000 HIV-negative adults at high risk of HIV infection (ClinicalTrials.gov Identifier: NCT00095576) [47]. Participants were randomized to vaccine or placebo and followed for incident HIV infection. Vaccine recipients were given three IM doses of 1.5×10^10^ virus genomes at weeks 0, 4, and 26. All participants signed informed consent and the following list of institutional human subjects review committees approved the protocol prior to study initiation: Universidade Federal do Rio de Janeiro Ethics Committee, STD/AIDS Reference and Training Center Research Ethics Committee, Instituto Dermatologico & Cirugia de Piel Ethics Committee, Human Rights Committee of the GHESKIO Centers, Cornell University IRB, Vanderbilt University IRB, University of the West Indies Ethics Committee, IMPACTA Ethics Committee, University of Puerto Rico IRB, University of Alabama IRB, University of Chicago IRB, New York Blood Center IRB, Columbia University IRB, Partners Human Research Committee, Fenway Community Health IRB, Vanderbilt University IRB, University of Pennsylvania IRB, University of Rochester Research Subjects Review Board, St Louis University IRB, Fred Hutchinson Cancer Research Center IRB, and UCSF Committee on Human Research.

Participants selected for the epitope mapping study cohort (n = 20) received all 3 injections, were HIV-1 uninfected throughout the follow-up period, and had peripheral blood mononuclear cell (PBMC) samples available from the Week 8 (post-enrollment) time point (Primary immunogenicity time point, 4 weeks after the second vaccination). Participants were stratified based on either a positive (n = 15) or negative (n = 5) T-cell response in a previous IFN-γ ELISpot assay at the same time point. We also included 5 placebo recipients as negative controls. All participants were previously typed for their HLA class I alleles using sequence-based typing.

### ELISpot assay and epitope mapping strategies

Validated IFN-γ ELISpot assays were performed on previously cryopreserved PBMCs using previously detailed methods [48]. Upon thawing, the PBMC viability in all samples was above 80%, and the majority were above 90% after an overnight rest. Briefly, 100,000 *ex vivo* PBMCs were stimulated overnight in triplicate for all test conditions in the presence of individual or pools of peptides at a final concentration of 1 μg/mL each. Peptides and pools were initially reconstituted in DMSO (50 mg/mL) from individually lyophilized 15-mer peptides. For each participant, 6 negative control wells (PBMCs with media alone) and 3 positive control wells (PBMCs incubated with PHA) were also tested. Responses are reported in spot-forming cells per million (SFC/M) as the mean of three replicate wells minus the mean of six replicate negative control wells.

Three epitope mapping strategies were evaluated concurrently: 1) “Test all”, 2) “Mini-pool”, and 3) “Matrix-pool” (details available in S1 File). ELISpot assays were performed using individual and pooled peptides, all 15 amino acids in length and overlapping by 11 amino acids; these were taken from a panel of 134 15-mers spanning the HIV-1 Gag Consensus B protein (Table A in S1 File; Gag ConB peptides, BioSyn; GenBank accession: AAS19377.1). The consensus sequence differed from the vaccine insert sequence at 7 amino acid residues. None of these differences were in peptides that elicited responses.

### Epitope identification

Participants often responded to two overlapping 15-mers, though the responses were likely specific to a single underlying epitope contained within the overlap. To determine the minimal set of epitopes that best accounted for the observed responses we applied the following criteria: if two 15-mers share a region of at least 11 residues then they can be explained by a single epitope, and that epitope is determined to be the entire region of overlap. Rarely, multiple consecutive overlapping 15-mers elicited responses. For this case epitopes were determined by iteratively applying the overlap criteria for each pair of overlapping peptides.

### Statistical analysis

All summary statistics are reported either with the standard error or the 95% confidence interval as noted by either a ± or “[LL, UL]”, respectively. Standard errors, confidence intervals and P-values were computed using bootstrap methods with participants as the independent unit of analysis, providing valid intervals and inference that account for within-vaccinee correlations of multiple assay readouts. Correlations were computed using Spearman’s rank correlation ρ. Wilcoxon rank sum tests were used for the comparison of two independent groups of response magnitudes, while signed-rank tests were used for comparing two groups of paired samples. All data available in *Supporting Information* (S2 File).

## Results

### Vaccine-primed T cells recognize HIV-1 Gag in master peptide pools

We selected 20 Step study participants [47] who received all 3 injections of the Ad5-vectored Gag/Pol/Nef HIV-1 candidate vaccine; 15 had a positive and 5 had a negative response in a prior IFN-γ ELISpot assay evaluating cryopreserved PBMC and using a pool of overlapping peptides spanning the Gag protein. Five placebo recipient samples served as negative controls. T-cell responses four weeks after the second vaccination, were measured to both individual and pooled Gag 15-mers. Assay positivity was determined using MIMOSA [18], a statistical test that controls the false-discovery rate (FDR) at 0.1% using assay replicates and negative controls.

The largest peptide pool, the Gag “master” pool, contained 134 15-mers spanning the entire Gag protein. As anticipated, the master pool elicited Gag-specific T-cell responses in all 15 vaccine responders, with a mean magnitude of 571 ± 205 SFC/M. The magnitudes of the master pool responses correlated strongly with the summed magnitude of all individual positive peptides within the pool (ρ = 0.92 [0.71, 0.99]), though the pool magnitude was consistently lower than that of the summed peptides (mean difference 694 ± 406 SFC/M, p = 0.018). No positive responses to the master pool were detected among placebo recipients or the five vaccine non-responders (n = 10, mean 17 ± 5 SFC/M). The master pool responses were used to differentiate responders from non-responders in all other analyses.

### T cells of vaccine recipients respond to individual 15-mer peptides

**Fig 1:**
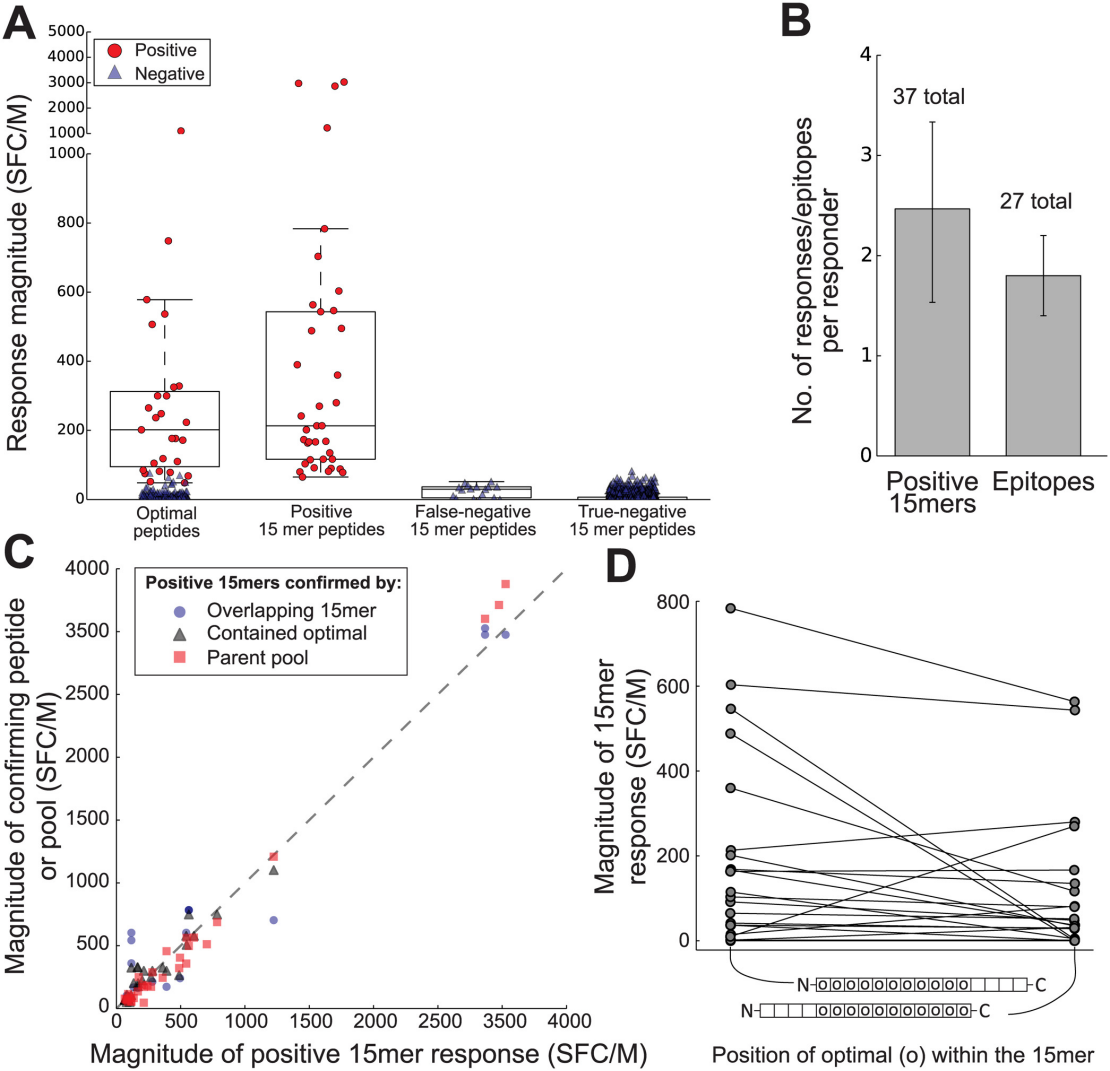
Vaccine primed IFN-γ ELISpot responses to HIV Gag peptides. Peptides derived from a consensus clade B Gag sequence were used individually to detect T-cell responses in participants (n = 15 vaccine responders) of the Step HIV vaccine trial. Responses were quantified in units of spot forming cells per million (SFC/M) using an IFN-γ ELISpot assay (A; one dot indicates the mean of a triplicate assay). Optimal length peptides were also used to further define the epitopes. Negative peptides containing the sequence of a positive optimal peptide were labeled as false-negative. Breadth was the minimal number of epitopes that explained the responses of each participant (B). Breadth was occasionally greater than the number of 15-mer responses when responses were detected to optimal peptides, but not the parent 15-mer peptide. The magnitudes of responses to 15-mer peptides and those that confirmed their positivity were plotted to assess their similarity and their correlation (C; dashes illustrate line of equality). Magnitude of response to a 15-mer peptide differed depending on the relative position of the epitope containing optimal peptide (D).

We assessed the responses of each participant to each of the 134 15-mers in the Gag master pool (Table A in S1 File), and detected 37 positive responses (mean magnitude 513 ± 209 SFC/M; Fig 1A). For positive responses the median coefficient of variation across replicates was 0.19 [0.13, 0.25]. This is equivalent to an 82 SFC/M standard deviation for a 500 SFC/M response. Among the responders, the mean number of responses was 2.5 ± 0.5 (Fig 1B). There were no positive responses to individual peptides among the placebo recipients or the vaccine non-responders.

Depending on sample availability, responders were tested using individual 8, 9, 10, 11, and 12 amino acid “optimal” Gag peptides previously found to elicit T-cell responses in HIV infected patients (Table B in S1 File). These were individually selected based on the restricting HLA allele and the HLA alleles of each responder. In total we tested 334 optimal peptides and detected 27 positive responses (Fig 1A). The magnitudes of responses to optimal peptides (293 ± 47 SFC/million) was not significantly different from those to 15-mers (p = 0.50).

To evaluate mapping strategies it was important to validate each response with at least one related response. The response was related either as: 1) an overlapping 15-mer sharing at least 8 amino acids, 2) an overlapping optimal peptide sharing at least 8 amino acids, or 3) a pool containing the peptide and no other positive peptides. Based on these criteria all 37 responses were validated. All but two responses were validated by overlapping peptides, suggesting that the epitopes were HLA class I restricted. The remaining two responses contained known HLA class I restricted epitopes. This is consistent with previous observations that while the Step vaccine primed both CD4+ and CD8+ T cells, CD8+ T cells were the primary producers of IFN-γ [30]. Given that 2010 total 15-mers were tested and all responses were validated, the false discovery rate is less than 0.05%, suggesting that we are using an appropriate (if slightly conservative) false-discovery rate cutoff for assay positivity.

Overall there was strong correlation between the magnitudes of responses to the positive 15-mers and those of the peptides or pools that validated them, including optimal peptides (Spearman’s ρ = 0.76 [0.49, 0.90]; n = 25), overlapping 15-mers (ρ = 0.75 [0.24, 0.98]; n = 32) and pools (ρ = 0.92 [0.72, 0.98]; n = 32) (Fig 1C). Additionally, the differences in magnitude between the responses to positive 15-mers and their validating pools or overlapping peptides were not significantly different from zero, suggesting that they were adequate proxies for the 15-mer response (Fig 1C, paired differences; optimals, mean 12 [–39, 53] SFC/M; pools, mean 19 [–86, 62] SFC/M).

### Responses to optimal peptides identify false negative 15-mer responses

We identified 17 15-mers which tested negative despite containing the sequence of a positive optimal peptide (0.8% of all negative 15-mer tests) (Fig 1A). Many of these negative 15-mers also overlapped positive 15-mers (n = 9) or were included in positive peptide pools that did not contain any other positive 15-mers (n = 5), thus meeting at least one of the other criteria that was used for validation of positive responses. Furthermore, their mean magnitude was 24 [17.5, 31.7] SFC/M, which is greater than negative assays overall (6.4 [5.0, 8.6] SFC/M). However, four of these putatively false negative 15-mers (two overlapping pairs each sharing an optimal) could not be confirmed by any other criteria, yet the response to the optimal peptide was well above background (78 and 537 SFC/M). In the subsequent evaluation of epitope mapping strategies, these responses were counted as two epitopes that were not detected by any of the strategies.

One possible explanation for a negative 15-mer that contains an optimal epitope is that the response depends on the location of the epitope within the 15-mer. To address this possibility, we examined the 22 optimal peptides that elicited positive responses and that were contained by two 15-mer parent peptides. Responses were significantly higher when the epitope was located toward the N-terminus compared to the C-terminus, with a mean difference of 77.5 SFC/M [17.6, 154.7, p = 0.014] SFC/M (Fig 1D).

### Underlying epitopes deduced from overlap of positive 15-mer and optimal peptides

We hypothesized that responses to overlapping peptides shared a single underlying epitope. Based on a simple set of criteria requiring at least 8 overlapping residues (see Materials and Methods for details) we determined that the 37 responses to 15-mers and the 27 responses to optimal peptides could be explained by 27 underlying epitopes. This corresponded to a breadth of 1.8 [1.4, 2.3] Gag epitopes per responder (Fig 1B). With the exception of two epitopes identified only by an optimal peptide, all of the epitopes could be confirmed by positive responses to at least two overlapping peptides. These 27 epitopes were used to evaluate the epitope mapping strategies.

### Small pools of peptides elicit robust T-cell responses

**Fig 2:**
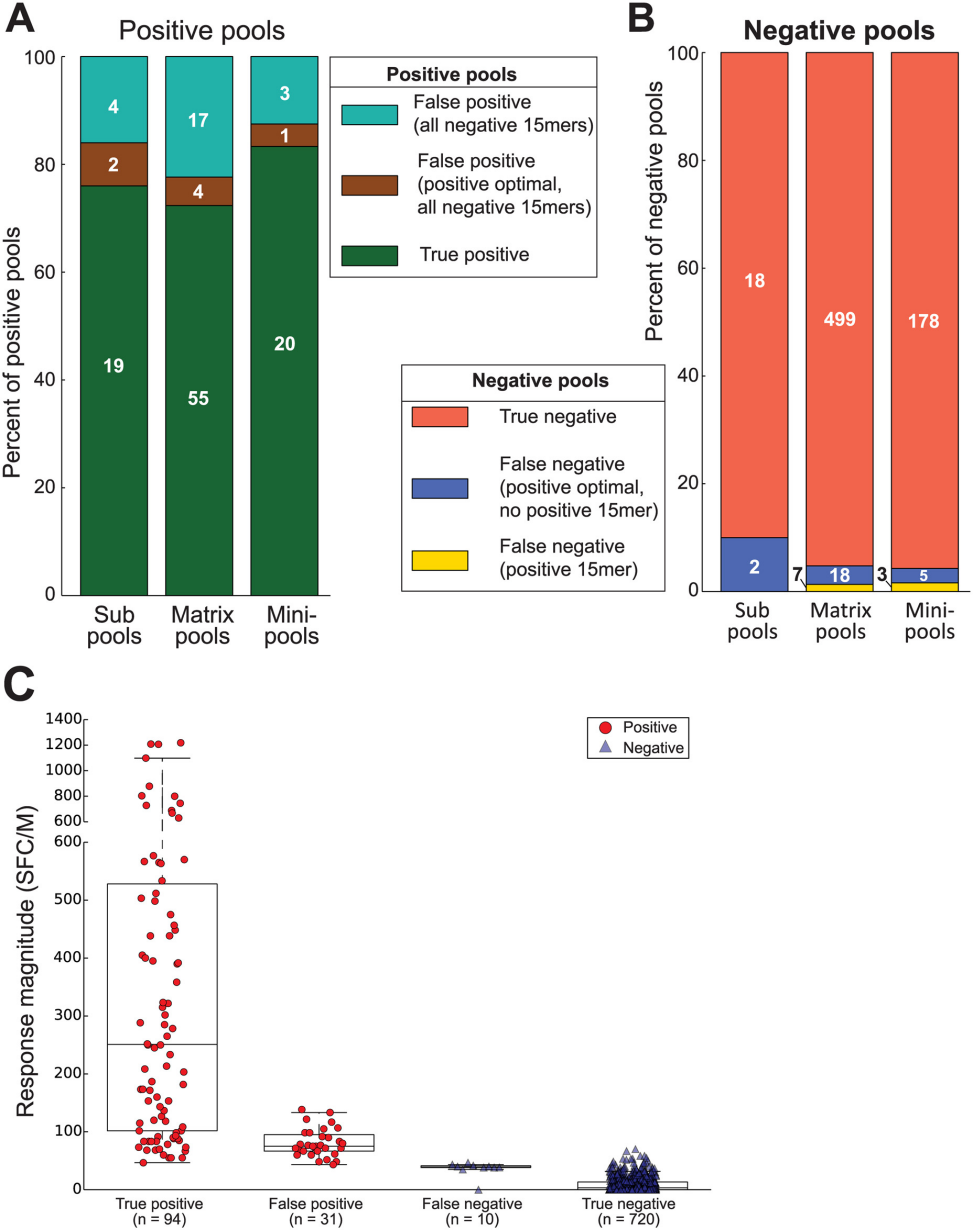
T-cell responses to small pools of 15-mer peptides. ELISpot responses to peptide pools were categorized based first on their positivity (A, positive; B, negative; C, response magnitudes, one dot indicates the mean of a triplicate assay) and subsequently on whether or not they contained a positive 15-mer or a 15-mer containing the sequence of a positive optimal peptide.

**Fig 3:**
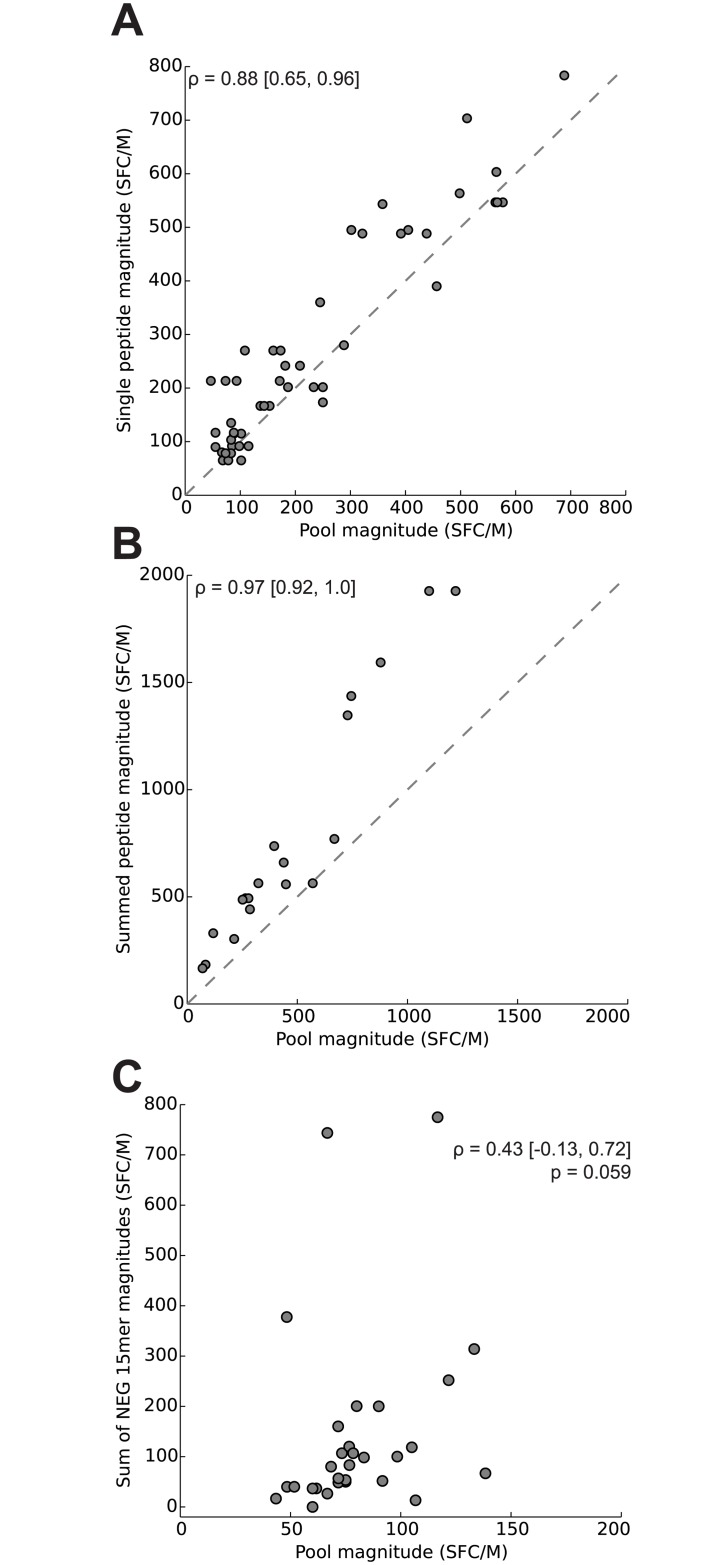
Correlation of responses to peptide pools and the peptides they contain. Rank-based correlations were assessed between the magnitudes of T-cell responses to positive peptide pools that contain a single positive peptide and the individual response to the positive peptide (A, one dot per pool, dashes indicate line of unity) and positive pools containing >1 positive peptide and the sum of the individual responses to the positive peptides they contain (B). Positive pools containing no positive 15-mer and >1 false negative 15-mer were plotted against the sum of the negative peptides they contained (C). 95% confidence intervals and p-value were computed using a participant-based bootstrap (n = 15 responders).

We tested for T-cell responses to pools of 15-mers that varied in their size and makeup. Each pool type was a stage in one of the epitope mapping strategies being evaluated. The positivity of responses to pools was determined using the MIMOSA method, controlling the false discovery rate at 0.1% within each pool type (i.e. master, sub, matrix and mini pools). There were no positive responses to any peptide pools among the placebo recipients and vaccine non-responders. Among the responders, 125 of 855 pools elicited a detectable positive response (Fig 2A and 2B). Of the positive pools, 75 ± 9% contained at least one 15-mer that elicited a positive response when tested individually, and as such were referred to as “true positive” pools (Fig 2A and 2C). The response magnitudes of the pools that contained a single positive peptide (n = 53 pools; mean 415 [196, 1044] SFC/M) were significantly lower than those of the single corresponding peptides (mean 443 [241, 966] SFC/M; paired difference, p < 0.001); however they were highly correlated (ρ = 0.88 [0.65, 0.96]) (Fig 3A). The magnitudes of the pools containing more than one positive peptide (21 pools; mean 744 [414, 1862]) were also significantly lower than the sum of the magnitudes of the positive peptides they contained (mean 1558 [680, 4949]; difference 814 [260, 2950] SFC/M, p < 0.001), though similarly they were also highly correlated (ρ = 0.97 [0.92, 1.0]; Fig 3B).

### Magnitudes of false positive pools correlate weakly with those of the negative 15-mers they contain

There were 31 positive pools whose peptides did not elicit responses when tested individually (Fig 2A). This corresponded to a false positive rate of 31/125 = 25 ± 9%. One possible explanation is that these putative false positive pools are due to decreasing assay specificity when pools of peptides are used for stimulation. Alternatively, ELISpot assays with pools of peptides may be more sensitive than those with single peptides, as they could summate multiple low magnitude responses. Several of these pools contained a 15-mer that assayed negative despite containing the sequence of a positive optimal peptide (i.e., false negative 15-mer). This may indicate that there were T-cell responses to an epitope that was not detected by the individual 15-mer, but was detected by the pool of 15-mers and by the optimal peptide. We plotted the magnitude of the response of each potentially false positive pool (n = 31; mean 81 [73, 91] SFC/M) against the sum of the magnitudes of the negative responses to the peptides contained by the pool (mean 144 [82, 283] SFC/M) (Fig 3C). The modest correlation (ρ = 0.43 [-0.13, 0.72], p = 0.059) also suggests that at least some of these pools may be detecting a response, possibly by summing multiple sub-threshold responses.

### False negative pools are the source of potential errors in epitope mapping strategies

The negative responses to pools of peptides were classified into two groups based on whether or not they contained a 15-mer that elicited a positive response (Fig 2B and 2C). Most negative pools did not contain a positive 15-mer (99% of all negative pools). However, 7 negative matrix-pools (1.3%), 3 negative mini-pools (1.6%) and zero negative sub-pools (0%) contained at least one positive 15-mer. These “false negative” pools were the source of errors in epitope mapping strategies. There are two possible explanations for the disagreement between the positive 15-mer and the negative pool containing that 15-mer: 1) the 15-mer elicits a response that is not detectable in the pooled peptide assay (e.g. peptide competition or antagonism in the pool); or 2) despite testing positive, neither the 15-mer nor the pool elicit true positive T-cell responses. Notably, the mean magnitude of these pools is greater than that of the other negative pools (36 [20, 41] SFC/M vs. 8.7 [6.4, 10] SFC/M, p < 0.001), indicating that there may be a signal that is below the threshold of detection. Indeed, the mean magnitude of the positive peptides in these negative pools (103 ± 12 [86, 135] SFC/M) is substantially lower than that of positive peptides overall (513 ± 191 [270, 1182] SFC/M), suggesting that these are low-level responses.

### Evaluation of the epitope mapping strategies

**Fig 4:**
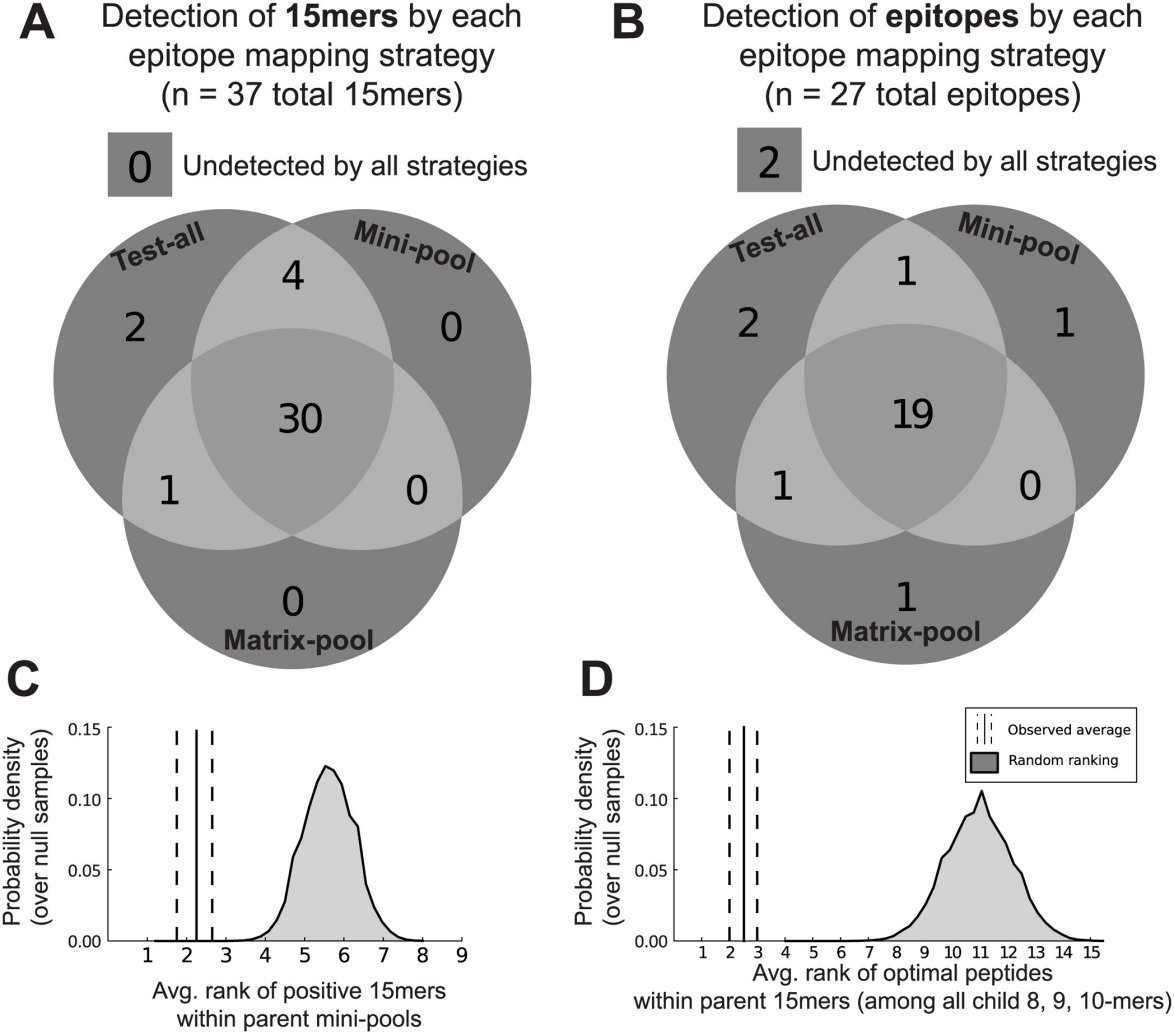
Evaluation of epitope mapping strategies. Epitope mapping strategies were assessed based on the number of positive 15-mers (A) and the number of epitopes (B) that were identified. By definition, all 15-mers were identified by the Test-all strategy. Epitopes detected using an optimal peptide were not necessarily identified by the Test-all strategy, but may have been detected by a pool strategy if all pools containing the epitope were positive. Potential benefits were explored of using computational HLA binding predictors to identify the epitope-containing 15-mer within a positive mini-pool (C, n = 20 pools) and to identify the optimal peptide within a positive 15-mer (D, n = 25 peptides). Ranks of the 15-mers in the mini-pool or the optimal peptides within the 15-mer were based on predicted HLA binding with each participant’s alleles. Observed average (line) and 95% confidence interval (dashed lines) are illustrated relative to a distribution of averages computed using random rankings.

With the results of the single peptide and pooled peptide ELISpot assays, we evaluated the efficiency and sensitivity of three T-cell epitope mapping strategies: 1) Test-all, 2) Mini-pool, and 3) Matrix-pool (details available in S1 File). The Test-all strategy is the least complex: for each participant, test for a response to each of the 134 Gag 15-mers individually. Despite the false negative 15-mers described above, the Test-all strategy successfully identified 23 of the 27 validated epitopes (Fig 4A and 4B). However, the Test-all strategy required the greatest number of assays– 134 single peptide assays per donor (Table 1).

**Table 1:** Number of assays required to identify epitopes using each of the strategies^†^.

	15-mer assays		Pool assays^‡^				
Strategy	Pos.	Neg.	Pos.	Neg.	Total assays (per ptcp)	Epitopes identified (per ptcp)	Assays per epitope
Test all	**37**	**1973**	**0**	**0**	**2010 (134)**	**23 (1.5)**	**89.3**
Mini pool	**34**	**200**	**24**	**186**	**444 (29.6)**	**21 (1.4)**	**21.1**
Matrix pool	**31**	**35**	**100**	**95**	**261 (17.4)**	**21 (1.4)**	**12.4**

^†^Includes only assays using samples from the 15 responders.

^‡^Includes only mini- and matrix-pools

The Mini-pool strategy is a two-staged, pooling approach in which 15-mer peptides are not tested individually if they fail to elicit a response as part of a pool of peptides. Since the mini-pool strategy depends on testing individual 15-mers in the final step, in practice it cannot identify any epitopes that are not also identified by the Test-all strategy (Fig 4A). However, in our evaluation of the strategy we included as successes those epitopes that were identified using optimal peptides and whose sequence was contained by a 15-mer within a positive mini pool, even if the parent 15-mer did not elicit a response (Fig 4B). By this measure, 6 epitopes went undetected by the mini-pool strategy, 2 of which were undetected by all strategies (including Test-all). The Mini-pool strategy required 29.6 ± 2 tests per participant, substantially lower than that for the Test-all approach.

The matrix-pool strategy is a three-staged, pooling approach which seeks to further reduce the number of necessary tests with a “matrix” of pools that identify the positive 15-mer in the final stage. This strategy successfully identified 21 of the 27 epitopes. Unidentified epitopes were the direct result of false-negative matrix pools–not false-negative sub-pools–as no false-negative sub-pools were observed. One epitope identified by an optimal peptide was also identified by intersecting matrix pools, but not by its parent 15-mer or sub-pool. Due to the efficient design of the matrix pools the strategy required significantly fewer tests than the mini-pool strategy (17.4 ± 3 tests per participant, n = 15 responders; p < 0.001).

Although the number of assays for each strategy is a good proxy for efficiency in general, the number of cryovials of PBMC required by each strategy is also relevant. In general, a single cryovial of PBMC can be used for a 96-well plate of ELISpot assays, though this can depend on cell recovery. A single plate allows for ~30 assays in triplicate in addition to negative and positive controls. Based on these numbers the Test-all strategy requires 5 cryovials per participant, independent of the number of responses. The Mini-pool strategy requires 1 cryovial for the pools in the first stage and 1 cryovial for the peptides in the second stage, per person. Theoretically more cryovials would be required if the response breadth was greater, but in this study all participants would have required two. The Matrix-pool strategy requires 1 cryovial for the sub-pools in the first stage, 1 or more cryovials for the matrix-pools in the second stage and 1 cryovial for the “intersection” peptides in the final stage. Therefore, based on this study, the Matrix-pool strategy would require at least one additional cryovial per participant and possibly two, compared to the Mini-pool strategy, depending on the number of positive sub-pools. However, since there are only three Gag subpools in the Matrix-pool strategy, this difference could be mitigated in practice by testing subpools for several HIV proteins on a single plate.

### HLA binding predictors could be used to further increase the efficiency of epitope mapping strategies

HLA binding predictors are computational tools that estimate the binding affinity of a short peptide with a specific HLA allele based on a large database of experimental data [49]. Though HLA binding is not the only factor that determines whether a vaccine peptide is targeted by T cells, predicted binding affinity has been used extensively to help identify vaccine-induced T-cell epitopes [50]. Given the class I HLA genotypes of the Step participants, we hypothesized that HLA binding prediction could be used to reduce the number of tests needed to identify the positive 15-mers in positive mini-pools. To assess the usefulness of HLA binding predictors for this purpose, we determined the binding affinity of each 15-mer, based on the minimum IC_50_ of all 8, 9 and 10-mers it contained with each of the participant’s four HLA-A and -B alleles (HLA-C and class II HLA alleles were excluded due to poor predictive performance). For each positive mini-pool, we determined the rank of the positive 15-mer in the pool. The ranks ranged from 1^st^ to 4^th^ with an average of 2.3 [1.8, 2.7] (Fig 4C). This demonstrated that within this cohort, a mini-pool strategy in which only the top 4 ranked peptides were tested within each mini-pool could have successfully identified all positive 15-mers and would have saved 144 negative 15-mer assays (9.6 per participant). As a comparison, we repeated the procedure 1,000 times assigning ranks randomly within each mini-pool. Under these conditions the average rankings of all the peptides formed a distribution centered at 5.5 (Fig 4C). None of the random iterations yielded a mean ranking that was as low as we observed, which provides a demonstration of the information added by the HLA binding predictions.

HLA binding predictors could also be used to identify an optimal epitope within a positive 15-mer [51]. To test this idea we used the 25 positive 15-mers, which contained the sequence of an optimal peptide that also tested positive, to assess the predictive performance of HLA binding predictors at identifying the optimal peptide within a 15-mer. For each 15-mer we ranked the optimal peptide and all 8, 9 and 10-mer peptides (21 *k-*mers) contained by the 15-mer, based on its minimum predicted IC_50_ across the responder’s four HLA-A and -B alleles. The rank of the optimal peptide among all *k*-mers ranged from 1^st^ to 5^th^ with an average ranking of 2.5 [2.0, 3.0] (Fig 4D). As a reference, to show the information added by using the HLA binding predictors, we repeated this procedure 1,000 times using random rankings of the peptides within each 15-mer (Fig 4D). This demonstrated that HLA binding predictors could be used to reliably identify optimal peptides restricted by HLA-A and HLA-B when 15-mer peptides have been used for epitope mapping. As the performance of HLA-Cw and class II HLA binding predictors improves, and with more rigorous validation of their application, these techniques could be used more broadly to increase the efficiency and accuracy of epitope mapping strategies in vaccine trials.

## Discussion

We have comprehensively evaluated two epitope mapping strategies that are commonly employed for the identification of vaccine-induced T-cell epitopes. Compared to a approach of testing all the overlapping 15-mer peptides in Gag, the matrix-pool and mini-pool strategies each identified all but three of the detectable T-cell epitopes, while benefitting from the efficiency gains of a group testing design. The estimated mean breadth for both of the group testing strategies was 1.4 epitopes per participant compared to a breadth of 1.5 estimated using the Test-all strategy. Though the Test-all strategy is simple in design, as it lacks peptide pools and conditional testing, the efficiency gains of the group testing strategies (4.5-fold and 7.7-fold for the mini-pool and matrix-pool strategies, respectively) make it possible to use epitope mapping as a variable in large HIV pathogenesis studies [52–55], and as an immunogenicity endpoint in vaccine efficacy trials [30,56,57] for which sample volume and lab resources may be limiting. The efficiency advantage of both pooling strategies over the Test-all strategy was also evident in the reduced usage of PBMC cryovials, although the difference between the two pooling strategies might not be significant in the context of mapping T-cell responses in multiple proteins. It might be possible to forego the sub-pool stage altogether, using a two-stage matrix-pool approach that would further reduce the number of assays and cryovial requirement. However, reduced assays and sample usage at the sub-pool level would increase assays and sample usage at the matrix pool level, as negative sub-pools help to screen-out peptides that do not contain epitopes. In theory, eliminating the sub-pool stage may also increase the sensitivity of the matrix-pool strategy, however in practice there were no false-negative sub-pools and therefore no responses went undetected due to the sub-pool level of assays.

The increased efficiency of group testing strategies may also be particularly important for identifying epitopes in the pathogens that cause TB and malaria as their proteomes are much larger than HIV and there is evidence that a T-cell response may be necessary for an effective vaccine [7,58–60]. An epitope mapping strategy could potentially be further optimized either for greater efficiency or higher sensitivity, for example, by decreasing or increasing, respectively, the number of technical replicates of each assay. The efficiency and sensitivity we observe is based on three replicates for all pooled and single peptide assays. Others have demonstrated increased efficiency in sample usage by using a cultured ELISpot assay [41]; though the expansion of antigen-specific T cells may increase an assay’s sensitivity for detecting a response, the response may not mimic the phenotype or functionality of an *ex vivo* response.

One complexity in this evaluation was that the T-cell epitopes were not known with absolute certainty; we were limited by the individual 15-mer assays. This issue was mitigated by validating the responses using a positive overlapping peptide or positive parent pool. With this approach we were able to validate the true positivity of all the 15-mer responses. However, there were several “false negative” 15-mers that did not elicit responses despite containing the sequence of an optimal peptide that did. It is possible there were additional false negative 15-mers that we did not identify because we did not exhaustively test all optimal peptides for all negative 15-mers.

A potential pitfall of any group testing procedure is that the pooled assay may be less sensitive to any single positive signal within the pool. In the context of natural HIV-1 infection Addo et al. showed that matrices of small pools of peptides (<12) had sensitivities relative to individual peptide testing that ranged from 93 to 97% [45]. In this study we identified 10 instances in which a pool did not elicit a response, despite containing at least one peptide that did individually. The impact of these false negative pools was that the matrix-pool and mini-pool strategies each failed to identify 6 T-cell epitopes. Relative to the test-all strategy, the sensitivity of the pooling strategies was 91% (21 of 23 epitopes; Fig 4B). Responses to the false negative pools tended to be higher in magnitude than to negative pools overall, which suggested that there may have been low-level responses that were below the level of detection for the assay. Smaller pool sizes may help to minimize the problem by reducing the likelihood for antigen competition for MHC binding. However, larger pools are also the source of efficiency gains, since negative pools eliminate the need for further testing of the peptides contained by the pool.

Our findings suggest that a benefit of pooled assays may be increased sensitivity in detecting low level responses. We hypothesize that an ELISpot assay with a pool of 15-mers will be more sensitive than an assay of any one of the peptides tested individually, if there are multiple 15-mers that stimulate different subsets of T cells. This is supported by the strong correlation between the magnitudes of pools containing more than one positive 15-mer with the summed magnitude of the positive peptides within the pool (ρ = 0.97; Fig 3B), which shows that pools summate multiple responses and suggests that the false positive pools may actually be detecting low-level responses. Of the 31 putatively false positive pools, 7 included a 15-mer that contained the sequence of a positive optimal peptide, suggesting that there was indeed a T-cell response detected by the pooled assay (Fig 2A). Response summation is enabled by the design of pools that contain overlapping peptides, which also reduces the number of total tests required compared to other pool designs [43,44].

Our experiments support previous observations that 15-mers are not the ideal antigen for class I restricted T cells. Of the 22 positive optimal peptides that were contained by two parent 15-mer peptides, 15 had at least one parent that assayed negative. The position of an optimal peptide within a 15-mer has been shown to impact the magnitude of the response [61]; out data support this as well, although in contrast we find that the 15-mer magnitude is greater when the optimal is towards the N-terminus. In addition, when the 15-mer parent peptide did elicit a response, it was not significantly different from the response to the optimal peptide and the two were highly correlated (Fig 1C). Together this suggests that the impact of epitope location within a parent peptide may be epitope dependent. We suggest that it may be beneficial to increase the number of replicates for the assays of individual 15-mers, since this would help to increase the overall sensitivity to low level responses. The 19% coefficient of variation we observed is comparable to the 20% that was observed in another study of a validated ELISpot assay [62].

Though we have discussed strategies to detect T-cell epitopes near the limit of detection, some may question the protective or therapeutic benefits of such low-level responses. In the future it may be important to optimize for the efficient detection of only the robust immunodominant responses or for the detection of polyfunctional responses using dual-color ELISpot assays [62] or gene expression markers. Such optimization could allow for fewer tests. However, it may also be important to increase the sensitivity of the ELISpot assay, if only to reduce the minimum PBMC and peptide requirements. Until the sample requirements and sensitivities of other stimulated assays like ICS can better those of the ELISpot assay, it will remain the standard for mapping and quantifying vaccine-induced epitope specificities.

## Supporting Information

S1 File Supporting methods, tables and figures. (DOCX)

S2 File Tables of comma-separated values (CSV) containing ELISpot magnitudes and response calls. (CSV)
