# Supplementary material for: Pooled-Peptide Epitope Mapping Strategies Are Efficient and Highly Sensitive: An Evaluation of Methods for Identifying Human T Cell Epitope Specificities in Large-Scale HIV Vaccine Efficacy Trials
Source: PLoS One. 2016 Feb 10;11(2):e0147812. doi: 10.1371/journal.pone.0147812 (PMC4749288; doi:10.1371/journal.pone.0147812)
Supplement: S1 File — (DOCX) [file pone.0147812.s001.docx]

# Supplemental material

**Epitope mapping strategies**

### Three epitope mapping strategies were evaluated concurrently: 1) “Test all”, 2) “Mini-pool”, and 3) “Matrix-pool”. The objective of each strategy was to identify in each participant all vaccine-induced T-cell epitopes within the Gag protein. To achieve this, ELISpot assays were performed using individual and pooled peptides, all 15 amino acids in length and overlapping by 11 amino acids; these were taken from a panel of 134 15-mers spanning the HIV-1 Gag Consensus B protein (**Table A in S1**; Gag ConB peptides, BioSyn). In the strategies detailed below, each “test” refers to an ELISpot assay performed in triplicate. Positive responses were determined from the results of each test using the MIMOSA method (1), controlling the false-discovery rate (FDR) at 0.1%, across each assay type (i.e., individual, mini-pool, matrix-pool, sub-pool, optimal peptide). In all strategies a Gag “master pool” consisting of all 134 peptides is tested in the first stage, in parallel with other pools or peptides. The response to the master pool can be used to determine an individual’s positivity to the protein. Subsequent stages are not contingent on a positive master pool.

### *Test-all strategy*: In a single stage, each of the 134 peptides is tested individually for each participant (134 total tests per participant).

### *Mini-pool strategy* (**Fig A in S1**): In the first of two stages, the 15-mers are tested in 14 mini-pools, each containing 10 consecutive 15-mers (with the exception of Pool 14, which contains 4). For each positive mini-pool, mapping continues in a second stage in which each 15-mer contained within a positive mini-pool is tested individually.

### *Matrix-pool strategy* (**Fig A in S1**) (2): The first of 3 stages consists of 3 “sub-pools”, each containing either 45 or 44 15-mers. A positive sub-pool response leads to the testing of its associated “matrix-pools” in the second stage. The matrix-pools associated with each sub-pool consist of 12 or 14 pools, each containing 6 or 7 peptides, such that each 15-mer is contained by exactly 2 matrix-pools. These matrix-pools are designed such that each 15-mer can be uniquely identified by the “intersection” of two positive matrix-pools. In the third stage, the 15-mers at the intersections of the positive matrix-pools are tested individually.

### Typically, in a multi-staged, pooling-based mapping strategy, only the peptides within “positive” pools are tested in subsequent stages. However, for the purpose of this study, all potential tests were performed (e.g., all matrix-pools were tested without regard to the result of the parent sub-pool). Also, the final stage consists of testing the individual 15-mers contained in the positive pools, but to avoid unnecessary duplication each 15-mer was tested once (in triplicate) and the result was used in the evaluation of all three strategies.

### In addition to these three mapping strategies, we tested individual “optimal epitope” peptides depending on PBMC sample availability. The optimal epitopes were 8, 9, 10, 11 and 12-mers that have previously been mapped to represent epitopes restricted by defined HLA class I alleles (3) (**Table B in S1**). Optimal peptides were selected and prioritized based on each participant’s HLA class I alleles. Depending on available samples, first HLA-A restricted optimals were tested, followed by those restricted by HLA-B and HLA-Cw alleles.

# Figures

## Fig A. Illustration of the epitope mapping strategies. Peptide pool size and organization for the Mini-pool (A) and Matrix-pool (B) strategies.


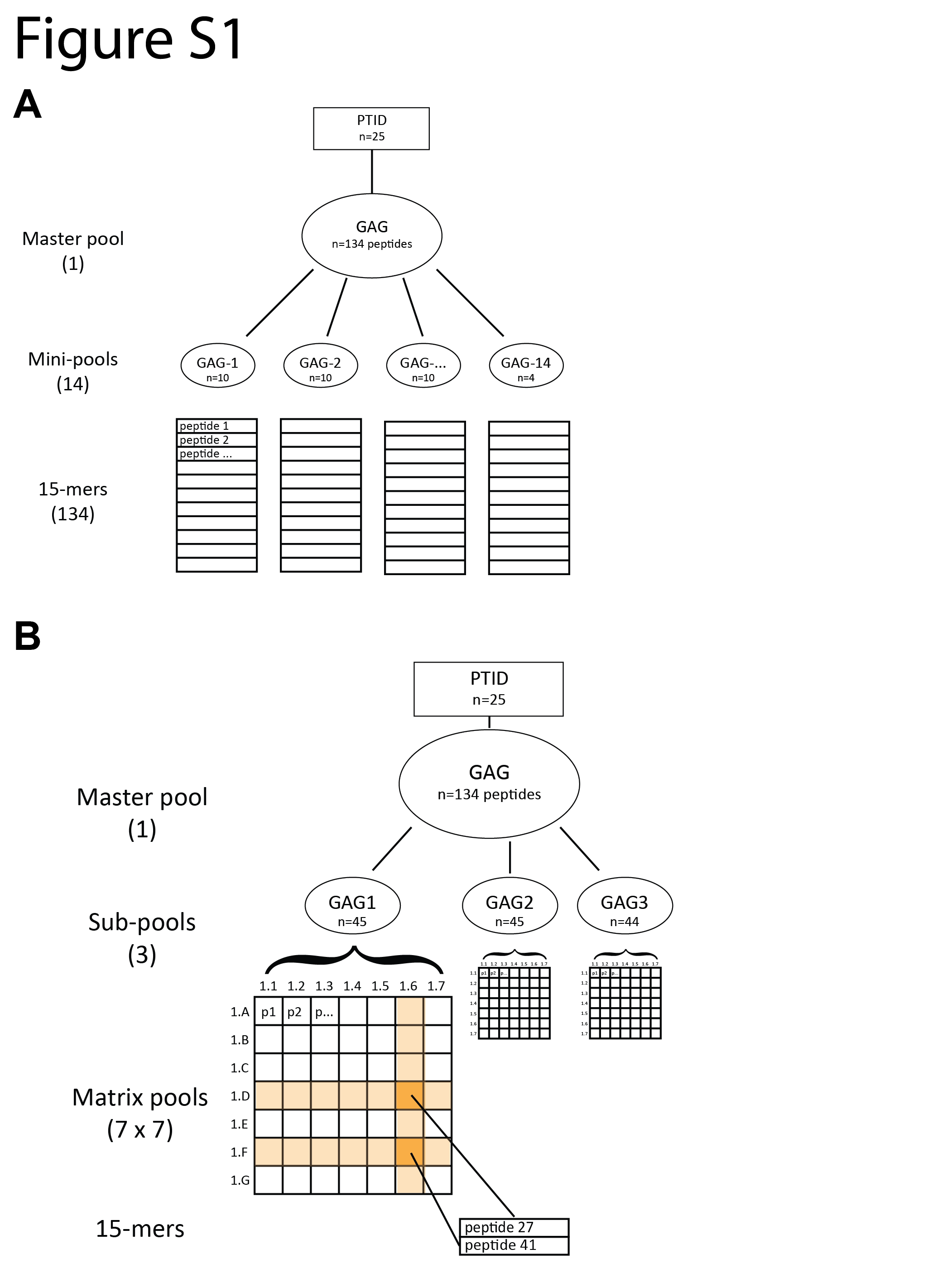


# Tables

## Table A. Gag 15-mer peptides.

| **Peptide #** | **Peptide ID** | **Sequence** | **AA Start** | **AA End** |
| --- | --- | --- | --- | --- |
| Gag ConB 1 | P501201 | MGARASVLSGGELDR | 1 | 15 |
| Gag ConB 2 | P501202 | ASVLSGGELDRWEKI | 5 | 19 |
| Gag ConB 3 | P501203 | SGGELDRWEKIRLR | 9 | 22 |
| Gag ConB 4 | P501204 | ELDRWEKIRLRPGGK | 12 | 26 |
| Gag ConB 5 | P501205 | WEKIRLRPGGKKKYK | 16 | 30 |
| Gag ConB 6 | P501206 | RLRPGGKKKYKLKHI | 20 | 34 |
| Gag ConB 7 | P501207 | GGKKKYKLKHIVWA | 24 | 37 |
| Gag ConB 8 | P501208 | KKYKLKHIVWASREL | 27 | 41 |
| Gag ConB 9 | P501209 | LKHIVWASRELERFA | 31 | 45 |
| Gag ConB 10 | P501210 | VWASRELERFAVNPGL | 35 | 50 |
| Gag ConB 11 | P501211 | ELERFAVNPGLLETS | 40 | 54 |
| Gag ConB 12 | P501212 | FAVNPGLLETSEGCR | 44 | 58 |
| Gag ConB 13 | P501213 | PGLLETSEGCRQIL | 48 | 61 |
| Gag ConB 14 | P501214 | LETSEGCRQILGQL | 51 | 64 |
| Gag ConB 15 | P501215 | SEGCRQILGQLQPSL | 54 | 68 |
| Gag ConB 16 | P501216 | RQILGQLQPSLQTGS | 58 | 72 |
| Gag ConB 17 | P501217 | GQLQPSLQTGSEELR | 62 | 76 |
| Gag ConB 18 | P501218 | PSLQTGSEELRSLY | 66 | 79 |
| Gag ConB 19 | P501219 | LQTGSEELRSLYNTV | 68 | 82 |
| Gag ConB 20 | P501220 | SEELRSLYNTVATLY | 72 | 86 |
| Gag ConB 21 | P501221 | RSLYNTVATLYCVH | 76 | 89 |
| Gag ConB 22 | P501222 | YNTVATLYCVHQRI | 79 | 92 |
| Gag ConB 23 | P501223 | VATLYCVHQRIEVK | 82 | 95 |
| Gag ConB 24 | P501224 | LYCVHQRIEVKDTK | 85 | 98 |
| Gag ConB 25 | P501225 | VHQRIEVKDTKEAL | 88 | 101 |
| Gag ConB 26 | P501226 | RIEVKDTKEALEKI | 91 | 104 |
| Gag ConB 27 | P501227 | VKDTKEALEKIEEEQNK | 94 | 110 |
| Gag ConB 28 | P501228 | ALEKIEEEQNKSKKK | 100 | 114 |
| Gag ConB 29 | P501229 | IEEEQNKSKKKAQQA | 104 | 118 |
| Gag ConB 30 | P501230 | EQNKSKKKAQQAAA | 107 | 120 |
| Gag ConB 31 | P501231 | KSKKKAQQAAADTGN | 110 | 124 |
| Gag ConB 32 | P501232 | KAQQAAADTGNSSQV | 114 | 128 |
| Gag ConB 33 | P501233 | AAADTGNSSQVSQNY | 118 | 132 |
| Gag ConB 34 | P501234 | TGNSSQVSQNYPIV | 122 | 3 |
| Gag ConB 35 | P501235 | SSQVSQNYPIVQNL | 125 | 6 |
| Gag ConB 36 | P501236 | VSQNYPIVQNLQGQM | 128 | 10 |
| Gag ConB 37 | P501237 | YPIVQNLQGQMVHQA | 132 | 14 |
| Gag ConB 38 | P501238 | VQNLQGQMVHQAI | 3 | 15 |
| Gag ConB 39 | P501239 | NLQGQMVHQAISPR | 5 | 18 |
| Gag ConB 40 | P501240 | GQMVHQAISPRTLNA | 8 | 22 |
| Gag ConB 41 | P501241 | HQAISPRTLNAWVKV | 12 | 26 |
| Gag ConB 42 | P501242 | SPRTLNAWVKVVEEK | 16 | 30 |
| Gag ConB 43 | P501243 | LNAWVKVVEEKAF | 20 | 32 |
| Gag ConB 44 | P501244 | AWVKVVEEKAFSPEV | 22 | 36 |
| Gag ConB 45 | P501245 | VVEEKAFSPEVIPMF | 26 | 40 |
| Gag ConB 46 | P501246 | KAFSPEVIPMFSAL | 30 | 43 |
| Gag ConB 47 | P500626 | SPEVIPMFSALSEGA | 33 | 47 |
| Gag ConB 48 | P501247 | IPMFSALSEGATPQDL | 37 | 52 |
| Gag ConB 49 | P501248 | ALSEGATPQDLNTML | 42 | 56 |
| Gag ConB 50 | P501249 | GATPQDLNTMLNTV | 46 | 59 |
| Gag ConB 51 | P501250 | PQDLNTMLNTVGGH | 49 | 62 |
| Gag ConB 52 | P501251 | LNTMLNTVGGHQAAM | 52 | 66 |
| Gag ConB 53 | P501252 | LNTVGGHQAAMQMLK | 56 | 70 |
| Gag ConB 54 | P501253 | GGHQAAMQMLKETI | 60 | 73 |
| Gag ConB 55 | P501254 | HQAAMQMLKETINEEA | 62 | 77 |
| Gag ConB 56 | P501255 | MQMLKETINEEAAEW | 66 | 80 |
| Gag ConB 57 | P501256 | KETINEEAAEWDRLH | 70 | 84 |
| Gag ConB 58 | P501257 | NEEAAEWDRLHPVHA | 74 | 88 |
| Gag ConB 59 | P501258 | AEWDRLHPVHAGPIA | 78 | 92 |
| Gag ConB 60 | P501259 | RLHPVHAGPIAPGQM | 82 | 96 |
| Gag ConB 61 | P501260 | VHAGPIAPGQMREPR | 86 | 100 |
| Gag ConB 62 | P501261 | PIAPGQMREPRGSDI | 90 | 104 |
| Gag ConB 63 | P501262 | GQMREPRGSDIAGTT | 94 | 108 |
| Gag ConB 64 | P501263 | EPRGSDIAGTTSTL | 98 | 111 |
| Gag ConB 65 | P500643 | GSDIAGTTSTLQEQI | 101 | 115 |
| Gag ConB 66 | P501264 | AGTTSTLQEQIGWM | 105 | 118 |
| Gag ConB 67 | P501265 | TSTLQEQIGWMTNNPPI | 108 | 124 |
| Gag ConB 68 | P501266 | EQIGWMTNNPPIPV | 113 | 126 |
| Gag ConB 69 | P501267 | GWMTNNPPIPVGEIY | 116 | 130 |
| Gag ConB 70 | P501268 | NNPPIPVGEIYKRWI | 120 | 134 |
| Gag ConB 71 | P501269 | IPVGEIYKRWIILGL | 124 | 138 |
| Gag ConB 72 | P501270 | EIYKRWIILGLNKIV | 128 | 142 |
| Gag ConB 73 | P501271 | RWIILGLNKIVRMY | 132 | 145 |
| Gag ConB 74 | P501272 | ILGLNKIVRMYSPTSI | 135 | 150 |
| Gag ConB 75 | P501273 | KIVRMYSPTSILDIR | 140 | 154 |
| Gag ConB 76 | P501274 | MYSPTSILDIRQGPK | 144 | 158 |
| Gag ConB 77 | P501275 | TSILDIRQGPKEPFR | 148 | 162 |
| Gag ConB 78 | P501276 | DIRQGPKEPFRDYV | 152 | 165 |
| Gag ConB 79 | P501277 | RQGPKEPFRDYVDRF | 154 | 168 |
| Gag ConB 80 | P501278 | KEPFRDYVDRFYKTL | 158 | 172 |
| Gag ConB 81 | P501279 | RDYVDRFYKTLRA | 162 | 174 |
| Gag ConB 82 | P501280 | YVDRFYKTLRAEQA | 164 | 177 |
| Gag ConB 83 | P501281 | RFYKTLRAEQASQEV | 167 | 181 |
| Gag ConB 84 | P501282 | TLRAEQASQEVKNWM | 171 | 185 |
| Gag ConB 85 | P501283 | EQASQEVKNWMTETL | 175 | 189 |
| Gag ConB 86 | P501284 | SQEVKNWMTETLLV | 178 | 191 |
| Gag ConB 87 | P501285 | VKNWMTETLLVQNA | 181 | 194 |
| Gag ConB 88 | P501286 | WMTETLLVQNANPDCK | 184 | 199 |
| Gag ConB 89 | P500665 | LLVQNANPDCKTILK | 189 | 203 |
| Gag ConB 90 | P501287 | NANPDCKTILKAL | 193 | 205 |
| Gag ConB 91 | P501288 | NPDCKTILKALGPAA | 195 | 209 |
| Gag ConB 92 | P501289 | KTILKALGPAATL | 199 | 211 |
| Gag ConB 93 | P500668 | ILKALGPAATLEEMM | 201 | 215 |
| Gag ConB 94 | P501290 | LGPAATLEEMMTA | 205 | 217 |
| Gag ConB 95 | P501291 | PAATLEEMMTACQGV | 207 | 221 |
| Gag ConB 96 | P501292 | LEEMMTACQGVGGPGH | 211 | 226 |
| Gag ConB 97 | P501293 | TACQGVGGPGHKARV | 216 | 230 |
| Gag ConB 98 | P501294 | GVGGPGHKARVLAEA | 220 | 3 |
| Gag ConB 99 | P501295 | PGHKARVLAEAMSQV | 224 | 7 |
| Gag ConB 100 | P501296 | ARVLAEAMSQVTNSA | 228 | 11 |
| Gag ConB 101 | P501297 | AEAMSQVTNSATIMM | 1 | 15 |
| Gag ConB 102 | P501298 | SQVTNSATIMMQR | 5 | 17 |
| Gag ConB 103 | P501299 | VTNSATIMMQRGNFR | 7 | 21 |
| Gag ConB 104 | P501300 | ATIMMQRGNFRNQRK | 11 | 25 |
| Gag ConB 105 | P501301 | MQRGNFRNQRKTVK | 15 | 28 |
| Gag ConB 106 | P501302 | GNFRNQRKTVKCF | 18 | 30 |
| Gag ConB 107 | P501303 | FRNQRKTVKCFNCGK | 20 | 34 |
| Gag ConB 108 | P501304 | RKTVKCFNCGKEGHI | 24 | 38 |
| Gag ConB 109 | P501305 | KCFNCGKEGHIAK | 28 | 40 |
| Gag ConB 110 | P501306 | FNCGKEGHIAKNCRA | 30 | 44 |
| Gag ConB 111 | P501307 | KEGHIAKNCRAPRKK | 34 | 48 |
| Gag ConB 112 | P500685 | IAKNCRAPRKKGCWK | 38 | 52 |
| Gag ConB 113 | P501308 | CRAPRKKGCWKCGK | 42 | 55 |
| Gag ConB 114 | P501309 | PRKKGCWKCGKEGH | 45 | 58 |
| Gag ConB 115 | P501310 | KGCWKCGKEGHQMK | 48 | 61 |
| Gag ConB 116 | P501311 | WKCGKEGHQMKDCTER | 51 | 66 |
| Gag ConB 117 | P501312 | EGHQMKDCTERQANF | 56 | 70 |
| Gag ConB 118 | P501313 | MKDCTERQANFLGKI | 60 | 74 |
| Gag ConB 119 | P501314 | TERQANFLGKIWPSH | 64 | 78 |
| Gag ConB 120 | P501315 | ANFLGKIWPSHKGR | 68 | 81 |
| Gag ConB 121 | P501316 | LGKIWPSHKGRPGNF | 71 | 85 |
| Gag ConB 122 | P501317 | WPSHKGRPGNFLQSR | 75 | 89 |
| Gag ConB 123 | P501318 | KGRPGNFLQSRPEPTA | 79 | 94 |
| Gag ConB 124 | P501319 | NFLQSRPEPTAPPEESF | 84 | 100 |
| Gag ConB 125 | P501320 | PEPTAPPEESFRF | 90 | 102 |
| Gag ConB 126 | P501321 | PTAPPEESFRFGEET | 92 | 106 |
| Gag ConB 127 | P501322 | PEESFRFGEETTTPSQK | 96 | 112 |
| Gag ConB 128 | P501323 | FGEETTTPSQKQEPI | 102 | 116 |
| Gag ConB 129 | P501324 | TTTPSQKQEPIDKEL | 106 | 120 |
| Gag ConB 130 | P500703 | SQKQEPIDKELYPLA | 110 | 124 |
| Gag ConB 131 | P501325 | EPIDKELYPLASLR | 114 | 127 |
| Gag ConB 132 | P501326 | DKELYPLASLRSLF | 117 | 130 |
| Gag ConB 133 | P501327 | LYPLASLRSLFGND | 120 | 133 |
| Gag ConB 134 | P501328 | LASLRSLFGNDPSSQ | 123 | 137 |

## Table B. Optimal epitope peptides (3).

| SLYNTVATL | CRAPRKKGC | MYSPTSIL | QVSQNYPIV | TERQANFL |
| --- | --- | --- | --- | --- |
| FLGKIWPSYK | RAEQASQEV | NYPIVQNL | EEAAEWDRV | AEWDRLHPV |
| VLAEAMSQV | EVIPMFSAL | ILGLNKIVR | NANPDCKTI | KETINEEAA |
| QAISPRTLNAW | DRFFKTLRA | TLYCVHQR | RMYSPTSI | GELDRWEKI |
| ETINEEAAEW | SPRTLNAWV | IILGLNKIVR | TSTVEEQIQW | EELRSLYNTV |
| ELRSLYNTV | VHPVHAGPIA | EIYKRWIIL | GQMVHQAISP | EPTAPPEESF |
| FRDYVDRFYK | TPQDLNTML | IRLRPGGKKK | TPQDLNQMLNTV | TPSQKQEPI |
| IRLRPGGKK | DRFYKTLRA | VIPMFSAL | ACQGVGGPGHK | EPIDKELYPL |
| KRWIILGLNK | YPLASLRSLF | ELDRWEKIRL | KYKLKHIVW | KIRLRPGGKK |
| ATLEEMMTA | HLVWASREL | RFAVNPGLL | RDYVDRFFKTL | TLYCVHQRI |
| MTNNPPIPV | TPQDLNTMLN | IATLWCVHQR | GLNKIVRMY | DCKTILKAL |
| TLNAWVKVV | DRFYKTRAE | FSPEVIPMF | GHQAAMQML | RDYVDRFYKTL |
| RMYSPTSIL | GPSHKARVL | HQAAMQMLK | IYKRWIIL | AEQASQDVKNW |
| YVDRFYKTL | LARNCRAPRK | TLRAEQATQD | IYKRWIILGL | EVKDTKEAL |
| EPFRDYVDRF | RLRPGGKKK | NSPTRREL | EEKAFSPEV | GGKKKYKKL |
| GGKKKYKL | RLRPGGKKKY | ISPRTLNAW | PQDLNTMLN | RSLYNTVATLY |
| GSEELRSLY | WASRELERF | KAFSPEVIPMF | GEIYKRWII | GPIAPGQM |
| NPDCKTIL | NSSKVSQNY | TSTLQEQIGW | ATQEVKNWM | SEGATPQDL |
| EIYKRWII | PPIPVGDIY | KELYPLTSL | VKNWMTETLL | GPGHKARVL |
| LSEGATPQDL | KIRLRPGGK | IEIKDTKEAL | QEPIDKELY |  |

**References**

1. **Finak G, McDavid A, Chattopadhyay P, Rosa SDE, Roederer M, Gottardo R**. 2013. Mixture Models for Single-Cell Assays with Application to Vaccine Studies. Biostatistics 1–10.

2. **Russell ND, Hudgens MG, Ha R, Havenar-Daughton C, McElrath MJ**. 2003. Moving to human immunodeficiency virus type 1 vaccine efficacy trials: defining T cell responses as potential correlates of immunity. J. Infect. Dis. **187**:226–42.

3. **Frahm N, Linde C, Brander C**. 2006. Identification of HIV-derived, HLA class I restricted CTL epitopes: insights into TCR repertoire, CTL escape and viral fitness. HIV Mol. Immunol. 3–28.
